# Whole-Genome Sequencing and Variant Analysis of Human Papillomavirus 16 Infections

**DOI:** 10.1128/JVI.00844-17

**Published:** 2017-09-12

**Authors:** Pascal van der Weele, Chris J. L. M. Meijer, Audrey J. King

**Affiliations:** a National Institute for Public Health and the Environment (RIVM), Centre for Infectious Disease Research, Diagnostics and Screening, Bilthoven, the Netherlands; b Vrije Universiteit-University Medical Center (VUmc), Department of Pathology, Amsterdam, the Netherlands; International Centre for Genetic Engineering and Biotechnology

**Keywords:** HPV16, genetic epidemiology, whole-genome sequencing

## Abstract

Human papillomavirus (HPV) is a strongly conserved DNA virus, high-risk types of which can cause cervical cancer in persistent infections. The most common type found in HPV-attributable cancer is HPV16, which can be subdivided into four lineages (A to D) with different carcinogenic properties. Studies have shown HPV16 sequence diversity in different geographical areas, but only limited information is available regarding HPV16 diversity within a population, especially at the whole-genome level. We analyzed HPV16 major variant diversity and conservation in persistent infections and performed a single nucleotide polymorphism (SNP) comparison between persistent and clearing infections. Materials were obtained in the Netherlands from a cohort study with longitudinal follow-up for up to 3 years. Our analysis shows a remarkably large variant diversity in the population. Whole-genome sequences were obtained for 57 persistent and 59 clearing HPV16 infections, resulting in 109 unique variants. Interestingly, persistent infections were completely conserved through time. One reinfection event was identified where the initial and follow-up samples clustered differently. Non-A1/A2 variants seemed to clear preferentially (*P* = 0.02). Our analysis shows that population-wide HPV16 sequence diversity is very large. In persistent infections, the HPV16 sequence was fully conserved. Sequencing can identify HPV16 reinfections, although occurrence is rare. SNP comparison identified no strongly acting effect of the viral genome affecting HPV16 infection clearance or persistence in up to 3 years of follow-up. These findings suggest the progression of an early HPV16 infection could be host related.

**IMPORTANCE** Human papillomavirus 16 (HPV16) is the predominant type found in cervical cancer. Progression of initial infection to cervical cancer has been linked to sequence properties; however, knowledge of variants circulating in European populations, especially with longitudinal follow-up, is limited. By sequencing a number of infections with known follow-up for up to 3 years, we gained initial insights into the genetic diversity of HPV16 and the effects of the viral genome on the persistence of infections. A SNP comparison between sequences obtained from clearing and persistent infections did not identify strongly acting DNA variations responsible for these infection outcomes. In addition, we identified an HPV16 reinfection event where sequencing of initial and follow-up samples showed different HPV16 variants. Based on conventional genotyping, this infection would incorrectly be considered a persistent HPV16 infection. In the context of vaccine efficacy and monitoring studies, such infections could potentially cause reduced reported efficacy or efficiency.

## INTRODUCTION

Human papillomavirus (HPV) infection is one of the most common sexually transmitted infections (STIs) worldwide (1) and the causative agent of cervical cancer (2). HPVs are highly conserved double-stranded DNA viruses that have codiverged with human populations for millennia (3). Most infections regress naturally, but high-risk HPV (hrHPV) infections that are not cleared by the host can cause cervical intraepithelial neoplasia (CIN) and cancer. Of all the hrHPV types, HPV16 is the most carcinogenic, causing over 60% of cervical cancers worldwide (4). Based on whole-genome sequence data, HPV16 can be subdivided into four lineages (A to D), with a different carcinogenic potential and geographical heritage attributed to each lineage (5, 6). Lineages differ by between 1.0 and 10% at the whole-genome nucleotide level and are further divided into sublineages if the nucleotide difference between two variants from the same lineage is 0.5 to 1.0% (7). Variants that match host ethnicity, in turn, have been associated with increased risk of persistence (8) and more recently with increased risk of CIN3+ (6, 8). Additionally, multiple HPV16 variant coinfections are common (5), although the role of minority variants in an infection is unknown. In this study, we focus on identifying the diversity of major HPV16 variants circulating in a Dutch population via Sanger whole-genome sequencing (WGS). Whole-genome sequence analysis could provide increased resolution over individual genes or genomic segments. Moreover, differences in phylogenetic clustering, single nucleotide polymorphism (SNP) locations, and participant ethnicity were compared for clearing and persistent infections in a longitudinal cohort study among young women (16 to 29 years old) in the Netherlands.

Information about occurrence rates of type-specific (TS) reinfection events could be relevant in a vaccine context where vaccine efficacy and efficiency are being reported based on conventional genotyping assays (9–11). A TS reinfection could be interpreted as a false-positive persistent HPV16 infection and possibly lead to reduced reported vaccine efficacy if based solely on conventional genotyping results. Therefore, we sequenced persistent HPV16 infections with longitudinal follow-up to discriminate between true persistent HPV16 infections and TS HPV16 variant reinfection events, which have previously been shown to occur (12).

## RESULTS

**FIG 1:**
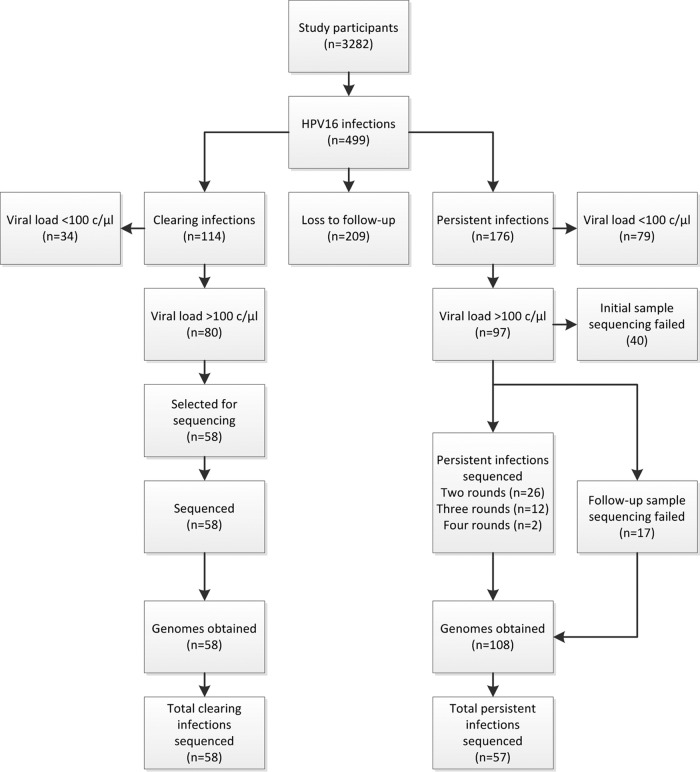
Schematic overview of selections made for persistent and clearing human papillomavirus 16 infections by duration (rounds), viral-load criterion, and sequencing results. Sequenced infections that persisted in three or four rounds had at least the initial and final samples sequenced. For clearing infections, 58 genomes were obtained from 58 infections; for persistent infections, 108 genomes were obtained from 57 infections.

In this study, 499 study participants (15.2%) were found to be HPV16 positive. Persistent infections were found in 176 participants (5.4%) (Fig. 1). Characteristics of the participant subsets included in this study are shown in Table 1, and the subsets were found to be representative of the respective total groups. Full genome sequences were initially obtained from 58 participants with clearing infections, resulting in 58 whole-genome sequences. Complete genomes were obtained from 57 participants with persistent infections. At least one round of follow-up was sequenced for 40 persistent infections, with an average of 70.3 weeks between the first and last available samples (minimum, 40 weeks; maximum, 148 weeks). An additional 17 persistent infections had only a single round sequenced, resulting in 108 genomes from persistent infections in total.

**TABLE 1:** Characteristics of the study and subsets of participants from whom complete HPV16 genomes were obtained^*a*^

Characteristic^*b*^	Value	
	Persistent infections sequenced (57/176)	Clearing infections sequenced (58/114)
Age (yr) [median (95% CI)]		
All persistent/clearing infections	25 (24–25)	23 (22–24)
Sequenced subset	24 (22–26)	22.5 (22–24)
C. trachomatis status (positive [*n*]/total [*n*])		
All persistent/clearing infections	17/176	16/114
Sequenced subset	4/57	5/58
European ethnicity (*n*)/total (*n*)		
All persistent/clearing infections	145/176	87/114
Sequenced subset	49/57	46/58
Mixed ethnicity (*n*)/total (*n*)		
All persistent/clearing infections	15/176	14/114
Sequenced subset	5/57	7/58
Asian ethnicity (*n*)/total (*n*)		
All persistent/clearing infections	10/176	11/114
Sequenced subset	3/57	5/58
Other ethnicities (*n*)/total (*n*)		
All persistent/clearing infections	6/176	2/114
Sequenced subset	0/57	0/58

a The selected subsets were not found to be significantly different from the total group for each parameter (*P* > 0.05).

b Differences in age were assessed by Student's *t* test, while differences in C. trachomatis status and ethnicity were assessed by Fisher's exact test. The distributions of Dutch and non-Dutch variants in clearing and persistent infections were also compared and found to be nonsignificant (Fisher's exact test; *P* = 0.29).

### Phylogeny.

**FIG 2:**
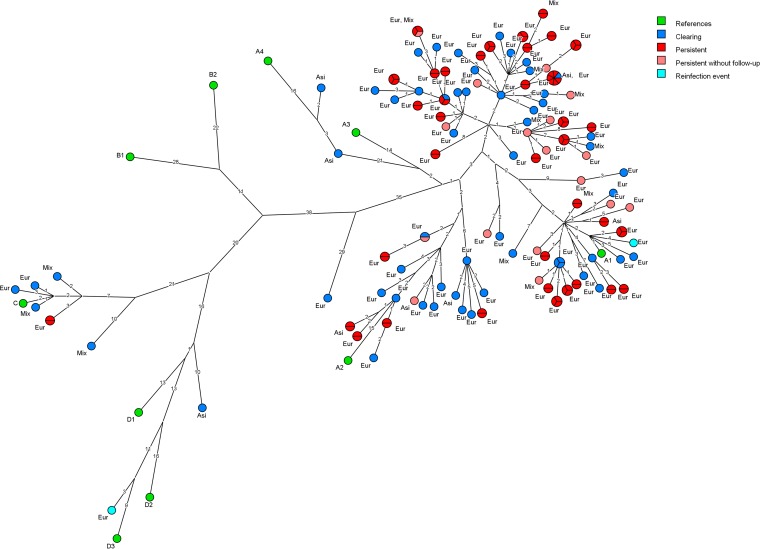
Maximum-parsimony tree for HPV16 sequences. Each circle represents a specific variant, while sections within the circle show how often a variant occurs. The reference strains, represented by green circles, are according to reference 7. Persistent infections are represented by red and pink circles, clearing infections by dark blue circles, and reinfections by light blue circles. Persistent infections had identical sequences at all sequenced time points. The reinfection event was initially considered a persistent infection based on conventional genotyping. The numbers on connecting lines indicate nucleotide differences between variants. The ethnicity of participants is indicated as follows: Eur, European; Asi, Asian; and Mix, mixed heritage.

Phylogenetic analysis of HPV16 genomes is shown in Fig. 2. Out of 115 HPV16 infections, 109 unique genomic variants were identified, meaning most infections were caused by unique sequence variants. Many of the identified variants differed from each other by less than 10 nucleotides (Fig. 2). The majority of study participants were infected with HPV16 genome variants clustering near reference A strains. A1 was the best-represented sublineage, with 79 variants. Sublineages A2 and A4 were represented by 19 and 2 variants, respectively, while sublineage A3 was not found within the data set. A subset of study participants were found to be infected with HPV16 variants representing strains C (*n* = 6), D1 (*n* = 1), and D3 (*n* = 1). No infections were found to cluster with lineage B. Upon sequencing, one participant with a clearing infection showed a variant that did not cluster with any of the described reference strains. The closest reference strain was found to be A3, with an 80-nucleotide difference. The high variant diversity was largely lost when zooming in on individual genes or the upstream regulatory region (URR), implying that variations occurred across the complete genome (data not shown). No clear difference could be identified between clearing and persistent infections based on phylogenetic comparison; however, participants infected with A4, C, and D strains (*n* = 10) seemed to clear the infections preferentially, as nine clearing infections and only one persistent infection were identified (*P* = 0.02).

### Ethnicity of participants.

The study participants were predominantly of European heritage, 86.0% (*n* = 49/57) and 79.7% (*n* = 47/59) among persistent and clearing infections, respectively (Table 1). Only 14.0% of persistent infections were in participants with non-European ethnicity (8.8% mixed [*n* = 5/57] and 5.2% Asian [*n* = 3/57]). Of the clearing infections, 19.3% were in non-European study participants (11.8% mixed [*n* = 7/59] and 8.5% Asian [*n* = 5/59]). No other ethnicities were reported in the sequenced subsets. The distribution of persistent and clearing infections was not affected by ethnicity for this study (Fisher's exact test; *P* = 0.62). Due to low numbers of non-European participants in the data set, statistical analysis matching variants with ethnicity was not possible.

### Longitudinal sampling of persistent infections.

Multiple whole-genome sequences were obtained from 40 study participants with persistent HPV16 infections. Twenty-six infections were sequenced at two sampling points, 12 at three points, and 2 at four points (Fig. 1). All but one sequence remained completely unchanged over time. One study participant, initially considered persistently infected, was actually found to have a different HPV16 variant in the follow-up sample, implying a type-specific reinfection. The initial sample clustered near reference strain A1, while the follow-up sample clustered near D3, with 151 nucleotide differences between samples (Fig. 2). Both samples from this study participant were resequenced and confirmed in an independent Illumina sequencing experiment (data not shown). Concordance between Sanger and Illumina consensus sequences was >99.8% for both samples.

### HPV16 WGS-based SNP analysis.

In total, 399 DNA SNPs, 12 insertions, and 7 deletions were identified compared to the reference strain, K02718, across study participants (data not shown). Of all SNPs, 136 (34.1%) were found to lead to amino acid changes (data not shown). None of the deletions or insertions were found in coding regions of the genome, except for one 63-nucleotide duplication in frame in E1, which had been described previously (13).

**FIG 3:**
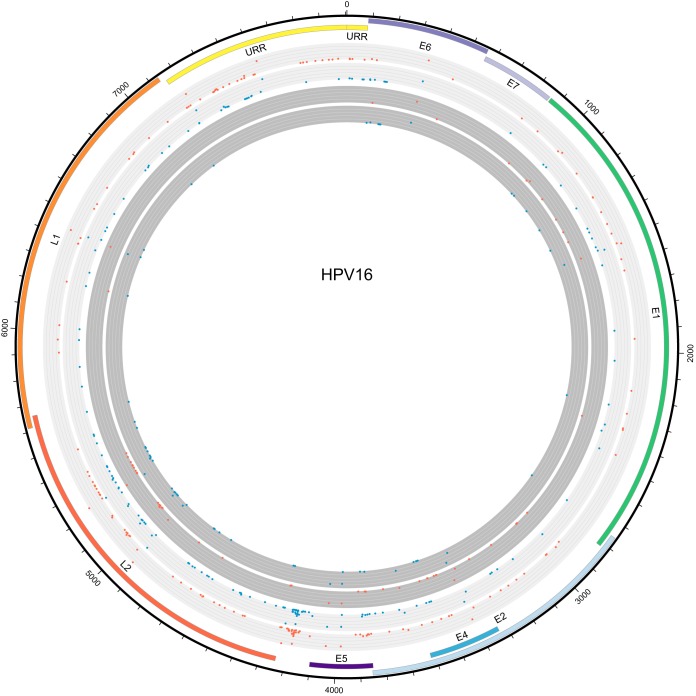
Circular representation of single nucleotide polymorphism comparison of the HPV16 genome. The light-gray circles show DNA variations, while the dark-gray circles show amino acid changes. Changes found among clearing (*n* = 50) and persistent (*n* = 55) variants are shown in blue and red, respectively. The heights of SNPs on the circles indicate relative incidences of variations in the data set compared to the reference strain, K02718.

**FIG 4:**
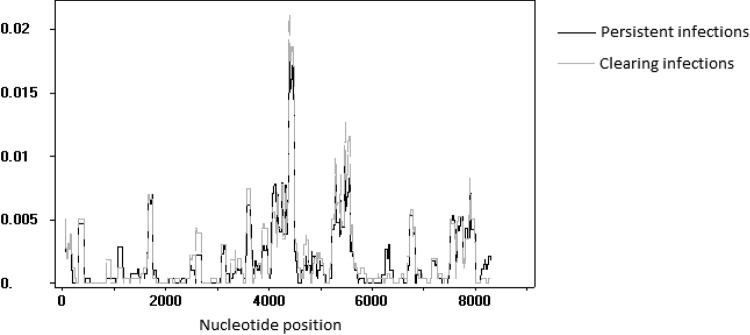
Plot of nucleotide diversity (pi) across the A1/A2 HPV16 genome based on sliding-window analysis. Data from persistent (*n* = 55) and clearing (*n* = 50) infections are represented in black and gray, respectively.

Including the above-mentioned reinfection event, the final data set consisted of 59 clearing and 56 persistent HPV16 infections. As non-A1/A2 variants were previously shown to clear preferentially, infections related to sublineages A1 and A2 were selected for SNP comparison. Non-A1/A2 strains were excluded from the analysis to prevent a bias in preferentially clearing SNPs from these variants. Participants infected with A1/A2-related strains (*n* = 105) were divided relatively evenly at 50 clearing and 55 persistent infections. SNP frequencies and comparisons between groups are shown in Fig. 3. No significant coding differences were found among participants leading to preferential persistence or clearing of infections. One noncoding SNP was found significantly more often among study participants with clearing infections than in those with persistent infections (*n* = 18 versus 8; *P* = 0.048), at position 4185 in the E5-L2 intergenic region. Sliding-window analysis showed similar patterns in nucleotide diversity between A1/A2 clearing and persistent infections (Fig. 4).

## DISCUSSION

In this study, we used whole-genome sequencing to reveal a remarkably large population of unique HPV16 variants circulating naturally in young women in the Netherlands. The observed diversity between HPV16 variants was large enough to allow us to discriminate between true persistent HPV16 infections and variant reinfections in a longitudinal cohort study. Additionally, preferential clearing of variants belonging to (sub)lineages A4, C, and D was observed. A large number of SNPs were identified in the present study compared to the reference strain, K02718; however, SNP diversity between clearing and persistent infections was nonsignificant for A1- and A2-related variants.

Phylogenetic analysis showed that most study participants were infected with variants clustering well with reference A lineages, as could be expected for a Dutch cohort (14). Among HPV16 genomes, one variant showed >1.0% (80 nucleotides) sequence difference with the phylogenetically closest reference strain (A3). Based on definitions introduced by Burk et al. (7), this variant could be considered a new lineage.

We found strong conservation of HPV16 variants within hosts with persistent HPV16 infections, indicating true persistence in the majority of infections. To our knowledge, we are the first to show that no sequence variation occurs in the complete genome of persistent infections with an average follow-up of 70.3 weeks between the initial and last available samples (minimum, 40 weeks; maximum, 148 weeks). Furthermore, we identified an HPV16 variant reinfection in one study participant where the initial sample clustered differently from the follow-up sample. This implies that conventional genotyping could lead to a false-positive observation of persistent infections, although considering the occurrence in this data set (1.8%; *n* = 1/57), conventional genotyping is quite adequate at classifying persistent HPV16 infections. In the context of vaccine efficacy, this could theoretically mean the difference between complete and partial reported protection, possibly justifying additional investigation in specific cases.

Despite the strong conservation of HPV16 sequences in persistent infections, we found that nearly all participants with HPV16 infections were infected with unique variants, although variation between variants could be as little as 1 nucleotide. Single nucleotide differences between variants could be due to sequencing errors. However, in persistent infections, we saw that variants were completely conserved through time, even with very small differences between related variants. This precludes the possibility that we found artificial variants due to sequencing errors.

When combined, variants belonging to (sub)lineages A4, C, and D seemed to clear preferentially compared to A1 and A2 variants (*P* = 0.02), but we could not perform additional statistics on combinations of variants and ethnicity due to the very limited number of study participants with non-European ethnicities. For A1- and A2-related variants, SNPs were assessed, as no phylogenetic distinction could be made between persistent and clearing infections. For these variants, clearance and persistence were relatively equally distributed. Over the complete data set, a large number of SNPs were identified, but only one noncoding SNP was significantly different between groups. This SNP has no known biologically relevant function. In addition, no clear difference was found between nucleotide diversities for clearing or persistent infections. This might imply that kinetics for early HPV16 infection are mostly host dependent, or at least less dependent on the viral genome. However, it could also mean that for the present study, the data set is too small to identify differences within phylogenetically related lineages. Our SNP comparison implies that any variations between infections are mere effects of chance under minimal evolutionary pressure for the data set. The source of this diversity might be ancient, considering papillomavirus evolution (3).

This study has a number of limitations. First, our definitions of persistent and clearing infections are not ideal. Due to the lack of information about the HPV status at baseline, clearing infections as defined in this study might potentially be a mixture of true incident and clearing persistent infections. In addition, we do not know the time during which persistent infections could have been present before they were detected in the present study or how long they would remain after the end of study. Therefore, we cannot exclude the possibility that the persistent infections we identified in this study ultimately cleared. This might explain why no strongly acting differences at the SNP level between persistent and clearing infections were identified. Unfortunately, because the study was designed as a chlamydia screening study, no cytologic or histologic follow-up is available. Therefore, we defined clearing infection as the apparent lack of HPV16 detection in follow-up samples. Our data do not discriminate between the various ways HPV infections could be cleared.

Although our data set shows great diversity among the population, it is relatively small for assessment of differences between clearing and persistent infections, especially if these effects are not very pronounced. Due to the study size, only very strong differences driving persistence or clearance of infection can be identified. In our data set, we do not identify a clear link between specific DNA variations and infection outcome, but it is possible that these effects are more nuanced for individual SNPs. Such effects would be beyond the capabilities of this data set, and further research on larger data sets is required to identify if these effects could drive persistence of HPV16 infection.

Sanger WGS was accomplished by amplification of two 4.5-kb fragments covering the full genome. A viral load of at least 100 copies (c)/μl was empirically determined as the minimum concentration of HPV from which full-length sequencing results could be expected. This could lead to a possible enrichment of variants resulting in high-viral-load infections.

Although Sanger sequencing is still the gold standard, it lacks the resolution generated by next-generation sequencing (NGS) techniques. Only the major variant driving the infection can be reliably determined, and any coinfections present in the samples cannot be reliably identified (15). On the other hand, major variants have been implicated in causing persistent infections, while minor variants appear more transient in nature (16). If a second variant is present in a sample at a concentration comparable to that of the major variant, Sanger sequencing could result in double peaks at certain nucleotide positions. In these instances, the software base-calling algorithm supplemented with manual verification was used to reach a definitive consensus. In this data set, double peaks were rare and generally limited to a single read, while other reads at the same position resulted in single clear curves.

Additionally, reinfections with the same variant could not be identified using our method. This could occur when the partner of the person sampled in this study carried a persistent infection, causing a “ping-pong” effect in which repeated exposure to the same variant occurs. It remains to be seen if even the increased resolution NGS provides could be of discriminative value for such cases. Further research utilizing NGS will be required to assess the role of minority variants in persistent infections.

For this study, no long-term follow-up is available. Study participants with infections that were identified as persistent might actually have slowly clearing infections. This could lead to a number of clearing infections in the persistent group, possibly impeding identification of SNPs truly associated with persistence of infection. Infections that were positive for two rounds followed by a negative sample were considered, but only two were identified in all of the sequenced data, preventing any analysis.

In summary, we applied whole-genome sequencing to show that HPV16 variants in the Netherlands are highly diverse between study participants but conserved through time in persistent infections. SNP analysis showed a large number of variable sites but no clear differences between clearing and persistent infections. This might imply that infection persistence at an early stage is weakly mediated by the virus and possibly more host related. Reinfection events can occur, albeit very rarely, in the population. In the context of vaccine efficiency studies, our results provide useful information about the behavior of HPV16 infections through time and may be of use in monitoring vaccine efficiency.

## MATERIALS AND METHODS

### CSI study design.

Vaginal self-swabs were collected from participants in the Chlamydia trachomatis Screening and Implementation Program (CSI). Study recruitment and methods have been described previously (17, 18). The 3,282 participants who gave additional consent for STI testing for organisms other than C. trachomatis and who answered a questionnaire were included in the study (19). Ethnicity was based on the country of birth of the study participants and their parents and was assigned according to the method of Woestenberg et al. (20). Ethnicity was divided into European, Asian, mixed (participants from the Caribbean and surrounding areas), and other ethnicities (combining all other nationalities reported for HPV16-positive study participants). Study participants supplied samples in up to four rounds each, with a median of 50 weeks between rounds (95% confidence interval [CI], 49 to 50 weeks; minimum, 5 weeks; maximum, 101 weeks). The study was approved by the Medical Ethical Committee of Vrije Universiteit-University Medical Center (VUMC), Amsterdam, the Netherlands (2007/239).

### HPV DNA detection, genotyping, and quantification.

Sample DNA isolation and HPV DNA genotyping have been described previously (19). Briefly, total DNA was extracted from 200 μl of vaginal swab using the MagNA Pure 96 platform (total nucleic acid isolation kit; Roche Diagnostics) according to the manufacturer's protocol and eluted in 100 μl. Genotyping was done using the SPF10-DEIA-LiPA_25_ platform (DDL Diagnostics) (21, 22). Samples positive for HPV16 were quantitated previously (23).

### Sample selection criteria.

An arbitrary PCR threshold was empirically defined at a viral load of 100 c/μl. Samples with HPV DNA concentrations below this value were considered likely to fail in the PCR step and were therefore not analyzed. Persistent HPV16 infections with the first and last samples above the viral-load threshold were selected for WGS analysis. For infections persisting for three or four rounds, at least the initial and last samples were sequenced. Persistent infections were defined as TS HPV16 positive in two or more sequential rounds with at least 40 weeks between samples. Infections with follow-up at less than 40 weeks were excluded (*n* = 1). In addition, to reach equal numbers of persistent and clearing infections, HPV16-positive samples that met the viral-load criteria were randomly selected from all participants with clearing HPV16 infections (Fig. 1). Clearing infections were defined as HPV16 positive in the round of sequencing, followed by an HPV16-negative test result.

### Long-template PCR and sequencing.

DNA eluates were subjected to long-template PCR to amplify the complete HPV16 genome. Two overlapping fragments encompassing the complete genome were generated using primer combinations F1832/R6382 and F6201/R2915 (reference 24 and data not shown). PCR was performed using TaKaRa PrimeStar GXL according to the manufacturer's protocol. The cycling conditions consisted of initial incubation at 98°C for 8 min followed by 38 cycles of 98°C denaturation for 15 s, 55°C annealing for 30 s, and 68°C elongation for 5 min and a final elongation step at 68°C for 15 min.

PCR product amplification was verified on the Lonza FlashGel system. If both fragments amplified successfully, samples were treated with ExoSap-It PCR product cleanup (Affymetrix) according to the manufacturer's protocol. If amplification failed for the initial sample, the follow-up sample was excluded from further analyses. If amplification succeeded for the initial sample but failed for the follow-up sample, the infections were sequenced without follow-up. Purified PCR products were subjected to Sanger sequencing using 45 unique primers for HPV16, covering the complete genome in both forward and reverse directions (references 24–28 and data not shown).

### Whole-genome sequencing and phylogenetic analyses.

The Sanger WGS data obtained were analyzed using CLC Genomics Workbench 9.5.3 (CLC Bio; Qiagen). For each sample, reads were assembled against the reference strain, K02718 (29). Assembled genomes with coverage of <1 at any nucleotide position were omitted from analysis. A consensus was generated based on assembled reads. The sequences obtained were verified manually in the assembly to compensate for possible base-calling errors by the software algorithm. Upon finalization of the consensus, sequences were exported to BioNumerics 7.2.5 (AppliedMaths) as GenBank (.gbk) files for phylogenetic analysis.

Reference strains used in phylogenetic analyses were selected based on the method of Burk et al. (7). The HPV16 lineages and sublineages represented were A1 to -4, B1 and -2, C, and D1 to -3.

If reinfection events were found within the longitudinal analysis of persistent infections, the initial sample of the infection was at that point regrouped under clearing infections for downstream analysis. The follow-up sample was treated accordingly depending on available further follow-up.

### SNP and statistical analysis.

For all samples, SNPs, amino acid changes, insertions, and deletions were analyzed with respect to the reference strain, K02718, using ProSeq 3.5. Coding regions for HPV16 genes were used according to Papilloma Virus Episteme (PaVE) (30; pave.niaid.nih.gov). SNP comparison was visualized using Circos (http://www.circos.ca). Nucleotide diversity (pi) between persistent and clearing infections was assessed using DNAsp with a sliding-window size of 100 and a step size of 1. Differences in individual SNP occurrences between clearing and persistent infections were assessed using Fisher's exact test. To compensate for possible sequence or interpretation errors, only SNPs occurring >1 time in the complete data set were considered for further investigation.

### Accession number(s).

The sequences obtained in this study were submitted to GenBank (accession numbers KY549156 to KY549321).
